# Three-Dimensional Accuracy of Facial Scan for Facial Deformities in Clinics: A New Evaluation Method for Facial Scanner Accuracy

**DOI:** 10.1371/journal.pone.0169402

**Published:** 2017-01-05

**Authors:** Yi-jiao Zhao, Yu-xue Xiong, Yong Wang

**Affiliations:** 1 National Engineering Laboratory for Digital and Material Technology of Stomatology, Beijing, PR China; 2 Research Center of Engineering and Technology for Digital Dentistry, Ministry of Health, Beijing, PR China; 3 Beijing Key Laboratory of Digital Stomatology, Beijing, PR China; 4 Center of Digital Dentistry, Peking University School and Hospital of Stomatology, Beijing, PR China; Virginia Commonwealth University, UNITED STATES

## Abstract

In this study, the practical accuracy (PA) of optical facial scanners for facial deformity patients in oral clinic was evaluated. Ten patients with a variety of facial deformities from oral clinical were included in the study. For each patient, a three-dimensional (3D) face model was acquired, via a high-accuracy industrial “line-laser” scanner (Faro), as the reference model and two test models were obtained, via a “stereophotography” (3dMD) and a “structured light” facial scanner (FaceScan) separately. Registration based on the iterative closest point (ICP) algorithm was executed to overlap the test models to reference models, and “3D error” as a new measurement indicator calculated by reverse engineering software (Geomagic Studio) was used to evaluate the 3D global and partial (upper, middle, and lower parts of face) PA of each facial scanner. The respective 3D accuracy of stereophotography and structured light facial scanners obtained for facial deformities was 0.58±0.11 mm and 0.57±0.07 mm. The 3D accuracy of different facial partitions was inconsistent; the middle face had the best performance. Although the PA of two facial scanners was lower than their nominal accuracy (NA), they all met the requirement for oral clinic use.

## Introduction

Facial morphology analysis is very important in craniofacial-maxillofacial surgery for preoperative diagnosis, postoperative evaluation, symmetry analysis, and so on. It can also give useful reference values for orthodontics, prosthodontics, and pedodontics. Facial morphology is currently a very active area of research. Conventional methods for facial appearance analysis are based on two-dimensional (2D) measurement methods, such as capturing series 2D photographs from different angles, and using Vernier caliper and bevel protractor to measure 2D projection distances and angles [1,2]. In recent years, with the development of optical scanning technology, facial morphology research has been raised to a new level from 2D to three-dimensional (3D) with the use of 3D facial scanners [3,4].

A 3D facial scanner is a non-contact optical measuring instrument that can acquire 3D facial models in open data format with real skin texture color, and a scanning process that is typically very short (less than one second). It is increasingly being reported in the literature that 3D facial scanners can be used in oral clinic, with the 3D facial models acquired by scanners being used for 3D quantitative diagnostic and treatment evaluation [4,5,6,7,8,9,10]. The accuracy of facial scanners in oral and maxillofacial clinical practice is a major focus of current research [11,12,13].

In general, a scanner has a nominal accuracy (NA) that is obtained by measuring standard geometry entities at the factory; a facial scanner is no exception. However, several recent studies have reported that there are indeed differences between NA and the practical accuracy (PA) of facial scanners, because the scanned object (a real person’s face) has a more complex shape and texture than a standard model [14,15]. In order to determine the level of differences between NA and PA, past studies used a contact measurement method to compare with the face scanning method [16,17]: using Vernier caliper and bevel protractor to measure the characteristic length and angles on real people’s faces, and comparing these values with the corresponding measurements on 3D facial models acquired by facial scanner in software. This evaluation method using contact measurement as the “Reference” (or Gold Standard) was very classical and commonly used in medical research [18]. However, it was difficult to avoid the touching deformation error of facial soft tissue for real persons. In addition, the limited number of characteristic lengths and angles in traditional methods can only represent part of the face feature—to the best of our knowledge, evaluation of the 3D global accuracy of whole facial scanning data has not been reported.

Moreover, it is obvious that oral clinical patients with facial deformities (such as jaw defects, edentulous jaw, malocclusion, and open bite) further increase the difficulty of data acquisition, the more complex the facial deformity, the lower the accuracy becomes. However, few studies have reported the PA of facial scanners for facial deformity patients.

This study evaluated the PA of optical facial scanners for facial deformity patients in oral clinic. Two typical kinds of facial scanning technologies—“Structured light” [19,20,21,22,23,24] and “Stereophotography” [25,26,27,28,29]—were included to evaluate and compare with each other. A “Line-laser” industrial scanner with high accuracy was adopted as the “Reference,” instead of the traditional contact measurement. Calculation of the 3D global and partial accuracy of facial scan was achieved by using “3D error” as a new measurement indicator.

## Materials and Methods

### 1 Experimental instrument

A variety of facial scanners are being used commercially and in the laboratory. However, their operating principle mainly includes “Structured light” and “Stereophotography,” which are two of the typical non-contact measurement technologies. The FaceScan system (Isravision, Darmstadt, GER) is a structured light scanner with a 0.2 mm NA. It can output OBJ format (a kind of triangulation model format) 3D face models with real color texture, and approximately 40 thousand triangles. The 3dMD Face system (3dMD, Atlanta, USA), which is based on stereophotography technology, has an NA of 0.2 mm. It can output WRL format (another kind of triangulation model format) 3D face models with real color texture, and approximately 60 thousand triangles. These two facial scanners were used in our experiment as the “Test” systems.

A “Line-laser” industrial scanner (Faro Edge LLP, Faro, Florida, USA) with a scanning principle as triangulation theory was used in this study as the “Reference” system. A Faro scanner is a flexible three-coordinate measuring arm with 0.059 mm NA (based on annual calibration certificate from Faro’s testing center, Shanghai, China), which is much higher than facial scanners. Its security Class II laser can sweep the skin surface continuously under the control of the operator and obtain feature data of the surface at the same time. Seven degrees of freedom makes the face scanning process an easy process that is completed in less than half a minute. The 3D face model acquired by Faro scanner is a point cloud model (with one face having approximately 100 thousand points with no texture), using reverse engineering software (Geomagic Studio 2012, 3D System, South Carolina, USA) to do post-processing—converting point cloud model to triangulation model, joining regional images together, eliminating the influence of human mobility, and saving in standard triangulation language (STL) format (a general 3D model format). The final face model comprises approximately 200 thousand triangles. Because the NA of the Faro scanner is much higher than that of our two test scanners, it was used as a reference to evaluate the other scanners.

### 2 Face model acquisition

Ten patients with different types of facial deformities, from tumor surgery, orthognathic surgery, orthodontics, and prosthodontics in Peking University School and Hospital of Stomatology were selected for this study during 2013.7–2015.7. The types of facial deformity included maxillary deficiency, mandibular excess, open bite, tumor resection, maxillary malocclusion, mandibular malocclusion, edentulous jaw, and jaw defect. All patients have provided informed consent and the individual in this manuscript has given written informed consent (as outlined in PLOS consent form) to publish these case details.

**Fig 1:**
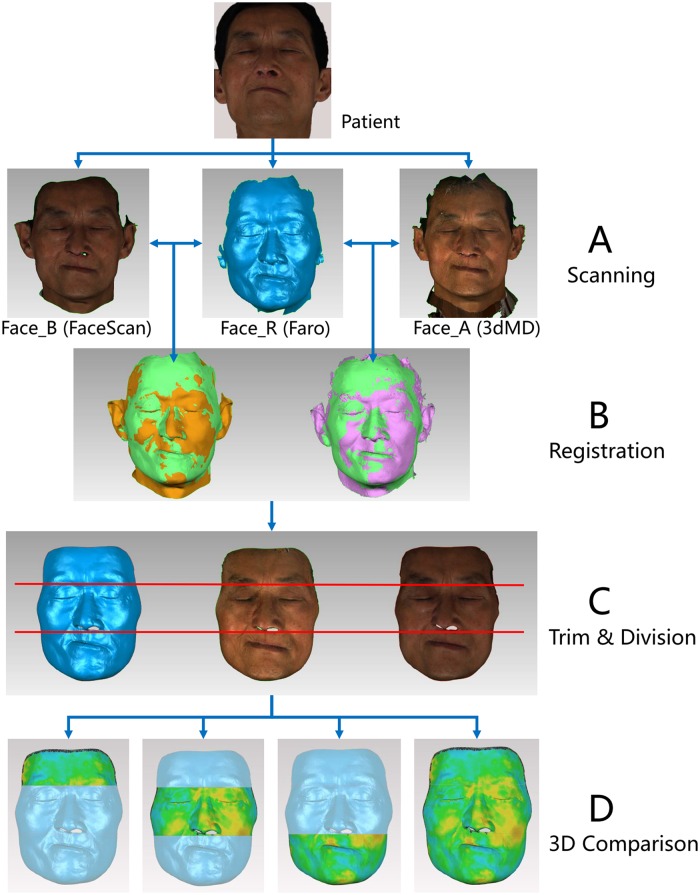
Experimental procedure. (A) Acquisition of face models (Face_A by 3dMD scanner, Face_B by FaceScan scanner, Face_R by Faro scanner); (B) Registration of reference and test models (green: Faro model, yellow: FaceScan model, purple: 3dMD model); (C) Trimming superimposed models by the same boundary and dividing into upper, middle, and lower parts. (D) 3D comparison between reference and test models in 3D error for partial and global accuracy of the test facial scanners.

For each patient, three face scanning operations were carried out, one for each of the reference scanner (Faro) and the two test scanners (3dMD and FaceScan) in one day. A reference 3D face model (Face_R) and two test models (Face_A and Face_B, A: 3dMD, B: FaceScan) were obtained (Fig 1A). In order to ensure that each patient maintained the same expression for the three scanning processes, the following measures were adopted: every patient was strictly controlled by the same researcher throughout the entire scanning process to maintain a sitting position, stable support for head and neck, natural head position (NHP), intercuspal position (ICP, a stable mandibular position), eyes and lips closing naturally, and relaxed body. The eyes were closed to reduce the impact of light projecting from the scanners on the patient’s expression. All 10 patients were scanned and 30 face models acquired. This study was approved by the IRB of Peking University School and Hospital of Stomatology (PKUSSIRB-2012046) and all participants signed an informed consent agreement.

### 3 Registration of face models

For each patient’s three face models, the “Registration” function of the 3D evaluation software (Geomagic Qualify 2012, 3D System, South Carolina, USA) was used to superimpose datasets in the following steps: (1) Set Face_R as a fixed model (the position should not be changed during the registration process); (2) Set Face_A as a floating model (the position could be changed during the registration process); (3) Appoint nine corresponding landmarks on fixed and floating model to primarily align the floating model overlapping Face_R; (4) Use the iterative closest point (ICP) algorithm of the software to further adjust the position of the floating model automatically and exactly overlapping Face_R. (5) Set Face_B as a floating model, then repeat Steps 3 and 4 (Fig 1B).

### 4 Trim and division of face models

In order to further reduce the influence on 3D accuracy analysis of the noise data on face models (such as hair, ear, and nostrils data), the three face models of each patient were trimmed in the same boundary using Geomagic Qualify software (Fig 1C). The appearance of hair is usually unstable and the ear area is the area most easily affected by hair. The area of the nostrils generally cannot be scanned because of the defect of optical scanning technology. These areas always have a large scanning error that is not attributable to the capability of the scanner. Furthermore, considering the little attention that has been paid to these areas, in our research, we removed these areas in order to purify the data and reserved overlapping regions in each group for accuracy analysis.

**Fig 2:**
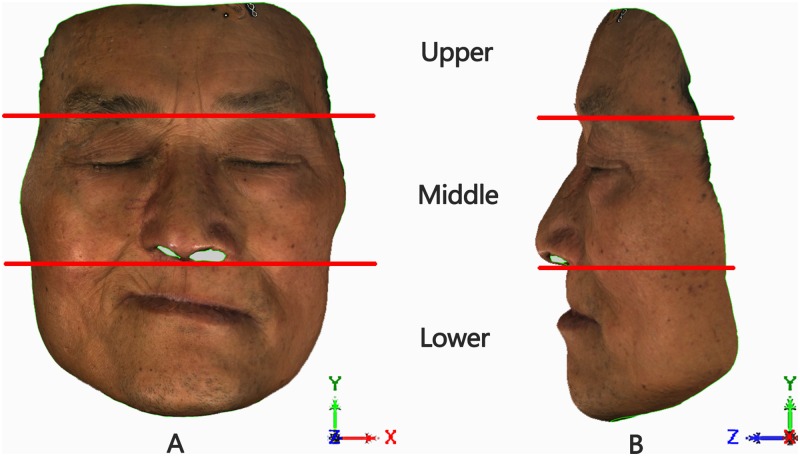
Division of face model for a maxillary defects patient. Patient’s head in the coordinate system (XYZ) of natural head position (NHP); the “red lines” were the cutting planes parallel to the XOZ system plane to segment the face model into upper, middle, and lower parts. (A) Front view of the face model; (B) Side view of the face model.

Then, the face models of each patient were divided into three parts using the Qualify software (Fig 2): (1) Lock the face model in NHP; (2) The “Upper” part, defined as the part of the face above the glabella plane (Face_RU, Face_AU, Face_BU); (3) The “Middle” part, from the glabella plane to the subnasale plane (Face_RM, Face_AM, Face_BM); (4) The “Lower” part, below the subnasale plane (Face_RL, Face_AL, Face_BL).

### 5 Three-dimensional dataset evaluation

The “deviation analysis” function in the Geomagic Qualify software was used to perform 3D comparison between the reference (Face_R) and test models (Face_A and Face B). For each patient, comparisons were conducted eight times to analyze the 3D global and partial deviation between facial datasets separately: Face_R was compared to Face_A and Face_B; Face_RU was compared to Face_AU and Face_BU; Face_RM was compared to Face_AM and Face_BM; Face_RL was compared to Face_AL and Face_BL (Fig 1D).

Color difference images were output to examine the congruency of reference and test models qualitatively. The “3D error” between each pair, defined as the root mean square (RMS) of all the distances between the closest point pairs on the reference and the test model, was calculated. The closest point pairs were searched and matched automatically by the algorithm of the software. The value of the RMS was calculated using the following formula:

$$RMS=\sqrt{\frac{\sum_{i=1}^{N}X_{i}^{2}}{N}}=\sqrt{\frac{X_{1}^{2}+X_{2}^{2}+\cdots +X_{N}^{2}}{N}}$$

If a point P_i_ on the reference model has a closest point P_i_’ on the test model, then X_i_ is the distance between P_i_ and P_i_’, and N is the total number of point pairs on both models. The 3D error (RMS) can serve as a measurement indicator of how far deviations between two different datasets vary from zero. In this study, low RMS scores, indicating a high 3D congruency of the superimposed models, signified good test model 3D accuracy.

The 3D errors of facial models for the 10 patients (each patient with eight comparisons) were finally calculated. The result was divided into the 3dMD group and the FaceScan group, and each group divided into four sub-groups as follows: The 3dMD group included Face_R to Face_A (called Group_A), Face_RU to Face_AU (Group_AU), Face_RM to Face_AM (Group_AM), and Face_RL to Face_AL (Group_AL). The FaceScan group included Face_R to Face_B (Group_B), Face_RU to Face_BU (Group_BU), Face_RM to Face_BM (Group_BM), and Face_RL to Face_BL (Group_BL). Each sub-group had 10 values (10 patients).

### 6 Statistical analysis

Statistical analysis was conducted using SPSS software (IBM SPSS Statistics 19.0, SPSS Inc., Chicago, USA). A K-S normality test was conducted for 3D errors (eight groups respectively) to exam the distribution of data (10 calculated values for each group). The mean and standard deviation (SD) of each group were calculated to evaluate the 3D global and partial accuracy of each scanner.

One-way ANOVA analysis was performed among the 1/3 partial face groups of 3dMD (Group_AU, Group_AM, and Group_AL), and 1/3 partial face groups of FaceScan (Group_BU, Group_BM, and Group_BL) to exam whether differences in the average 3D error were statistically significant for different facial partitions of each scanner. Homogeneity of variance test was also performed. To verify the statistical significant differences between each pair of groups after initial testing, Tukey honestly and Dunnett’s T3 post hoc tests were performed for equal variances and unequal variances, respectively.

Paired t-test analysis was also conducted to compare the 3D error of Group_A and Group_B in order to evaluate the difference in the 3D global accuracy between the two test scanners. Another three paired t-tests were also carried out between Group_AU and Group_BU, Group_AM and Group_BM, and Group_AL and Group_BL, with the aim of evaluating the difference in the partial 3D accuracy between two test scanners. The statistical significance was set at P < 0.05.

## Results

**Fig 3:**
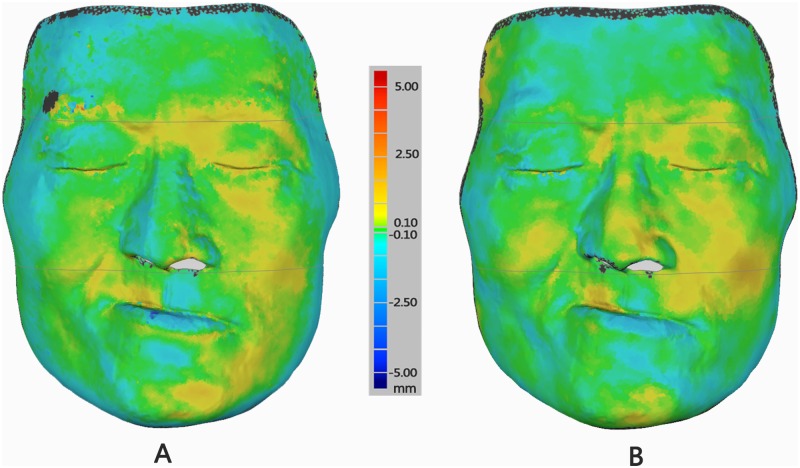
Difference color images of deviation between test and reference scanners for a maxillary defects patient. Difference color map is set from -5 mm to +5 mm. Yellow to red color indicates positive deviations, blue color shows negative deviation, green color signifies virtually zero error between two superimposed models. The black color shows the deviation out of 5 mm or -5 mm that would not be included in the calculation. (A) Difference color image of deviation between 3dMD (Test) and Faro (Ref) models; (B) Difference color image of deviation between FaceScan (Test) and Faro (Ref) models.

Color difference images (Fig 3) facilitated qualitative congruency analyses between test and reference models. The areas around the hair and eyebrows presented oversized deviation. By contrast, the middle of the face showed only minor deviation.

The K-S normality test for 3D errors (eight groups, 10 values per group) revealed that all groups conformed to normal distribution. Table 1 shows the mean (SD) of each data group. For 3dMD scanner, the middle face presented the highest 3D accuracy (least 3D error) of 0.48 ± 0.08 mm, and the upper face showed the highest accuracy 0.51 ± 0.14 mm for FaceScan scanner. Two test scanners for 10 facial deformities had a much closer 3D global accuracy: 3dMD of 0.58 ± 0.11 mm and FaceScan of 0.57 ± 0.07 mm. Comprehensively considering the value both of the mean and SD, the 3D accuracy of the middle of the face had the best performance among the three parts for the two test scanners.

**Table 1:** Mean (SD) of 3D error (in mm) for global and partial face models.

	3D error of 3dMD				3D error of FaceScan			
	Group_A	Group_AU	Group_AM	Group_AL	Group_B	Group_BU	Group_BM	Group_BL
Mean (SD)	0.58 (0.11)	0.55 (0.14)	0.48 (0.08)	0.67 (0.17)	0.57 (0.07)	0.51 (0.14)	0.52 (0.09)	0.64 (0.10)

SD: standard deviation

**Fig 4:**
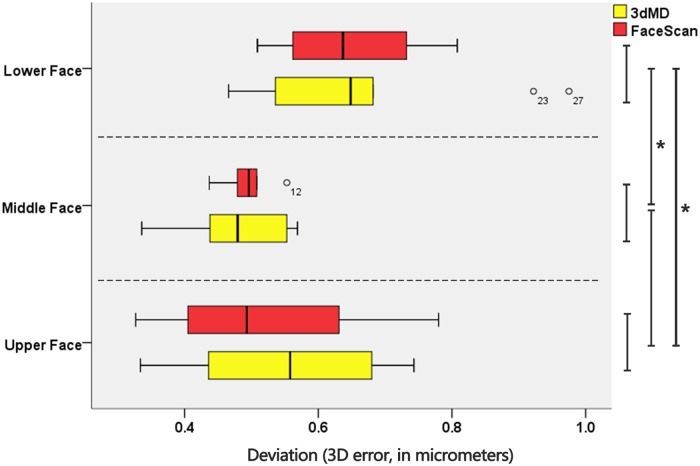
Boxplot of 3D error measurement for upper, middle, and lower face groups. The yellow boxplot shows data groups for 3dMD scanner, and the red for FaceScan scanner. The circles represent outliers, and asterisks signify P < 0.05.

Fig 4 shows the result of One-way ANOVA analysis among partial face (upper, middle, and lower) groups of 3dMD and FaceScan. Homogeneity of variance test showed an equal variance for all data groups. For the 3dMD scanner, Tukey honestly test showed no statistically significant differences (P > 0.05) in 3D error between upper and middle face, but statistically significant differences (P < 0.05) between lower—upper pair and lower—middle pair. The same was concluded for the FaceScan scanner. The 3D accuracy of the lower face for 3dMD and FaceScan were 0.67 ± 0.17 mm and 0.64 ± 0.10 mm, respectively (Table 1), which were lower than the value for upper and middle face.

Table 2 shows the result of paired t-test analysis of the 3D global and partial errors between the 3dMD and FaceScan scanners. Data analysis yielded no statistically significant differences (P > 0.05) in 3D error, whether upper, middle, nor lower (Fig 4), and also the global face between two test scanners. The results exhibited that there was no statistical difference between the 3D accuracy of 3dMD and FaceScan facial scanners.

**Table 2:** Paired t-test analysis result of 3D error between 3dMD and FaceScan scanners.

Paired Sample Group	P value of Paired t-test
Group_A and Group_B (Global)	0.896
Group_AU and Group_BU (Upper)	0.200
Group_AM and Group_BM (Middle)	0.343
Group_AL and Group_BL (Lower)	0.698

## Discussion

### 1 Superiority of the new facial scanner evaluation method—“3D error”

In previous studies, the contact measurement method is the most common method used to obtain the Reference (or Gold Standard) value in order to evaluate the accuracy of facial scanners. Liu et al. used Vernier calipers to measure 15 characteristic lengths on a plaster head model to evaluate the accuracy of one structured light facial scanner ((DSC-2, China) [16]. Zhao et al. used a coordinate measure machine (CMM) to acquire the coordinate values of 15 facial landmarks on a plaster head model to evaluate the accuracy of three different kinds of facial scanners [14]. The studies cited above are based on a steady measurement object, in this case, the plaster head model, which cannot effectively represent real patients in clinic. In order to evaluate a real situation, Xiong et al. acquired impressions of real patients’ face to make individual plaster face models. The individual face models were then used to evaluate the accuracy of a structured light facial scanner (TDOS, China) [30]. This method also uses contact measurement to obtain the reference value from the plaster model; however, it cannot avoid deformation error from the impression making processes. Ferrario et al. used a computerized electromagnetic digitizer to record facial landmarks directly by contacting an instrument stylus on the face of patients. The recording accuracy of this method ranged between 0.97 and 1.92 mm because of the soft-tissue deformation resulting from touching [18]. All of the methods above can only represent part of the face feature—a limited number of characteristic lengths and angles that cannot sufficiently represent the 3D shape of the global face.

Thus, in this study, we used a non-contacting 3D measurement system with a high accuracy of 0.059 mm as the reference system. This system could acquire a relatively accurate 3D reference face model for further 3D evaluation. The exclusive evaluation indicator for our method, “3D error,” can indicate the 3D shape congruency of the reference (from the reference system) and the test face model (from the test facial scanner). Compared with previous research, “3D error” is more comprehensive and more objectively reflects the 3D imaging capability of a facial scanner. The “3D error” algorithm involves all the data of the entire (or region) face model, so it can express more 3D shape information than only characteristic length and angle in traditional methods. Further, “3D error” is more significant for 3D morphological analysis and, to the best of our knowledge, has never been used to compare the accuracy of facial scanners.

### 2 Imaging mechanism of facial scanners and error cause analysis

Two typical facial scanners were evaluated in our study: the FaceScan system and the 3dMD system. The FaceScan system is a structured light scanner with an imaging mechanism based on triangulation measurement theory—a classic optical measuring algorithm. The charge coupled device (CCD) of the scanner acquires the deformation, caused by the changes in surface curvature, of the grating stripes projected onto a patient’s face. The computer algorithm analyzes the deformation and the structural parameters of the optical system to obtain the 3D shape of the face surface. The FaceScan system has a Double-Mirror structure that captures the three angles of 3D pictures simultaneously (0.2 s) and combines them to form one 3D face model. The 3dMD system is based on stereophotography technology. Stereophotography technology, also called binocular/multi-view stereovision, utilizes four cameras/CCD (two on each side) to acquire the depth information of the face surface from different angles in just 0.02 s. The computer algorithm it employs combines the four 3D pictures to form one 3D face model. The Faro system, a Line-laser scanner that is also based on triangulation measurement theory, is used as a reference system in our study because of its high NA (0.059 mm).

Ideally, a “zero error” reference system should be used to evaluate a facial scanner’s PA. However, because of the limits of the existing technology, this was not possible in this study. Structured light and stereophotography technology are the most commonly used facial scanning technology nowadays. The results of our previous research indicate that there is no statistically significant difference between these two technologies in terms of NA for plaster facial models [14] and also for normal face volunteers [15]. Consequently, we postulate that the error performance in different face regions was mainly caused by the surface deformity, with the expression consistency control having a small influence.

### 3 Three-dimensional accuracy of different facial partitions is inconsistent for facial deformity

The result of this study showed that, the scanning accuracy of Lower face had statistically significant differences with both the upper and middle face for two test facial scanners (Fig 4). The 3D accuracies of Lower face for 3dMD and FaceScan in this research were 0.67 (0.17) mm and 0.64 (0.10) mm. Compared with the values for upper and middle parts, they are obviously lower (Table 1). Two factors may account for these results: One is the influence of the scanner’s scanning ability; the other is the influence of the scanned object. We compared the scanning ability (3D accuracy) of two classic facial scanners. No difference appeared in any of upper or middle or lower face (Table 2). Thus, the first factor could be eliminated, and the variance was most likely because of the facial deformity.

We noticed that most of the patients included in this study had a facial deformity on the lower face, present as different levels of collapse and deflection. The unnatural shape of the facial tissue increased the undercut area, especially the labiofacial sulcus, oral fissure, and angle of mouth areas. The undercut area increases optical scanning difficulty, both for structured light and stereophotography, and also decreased the facial scan accuracy. The authors surmised that for more serious facial deformity, PA would be lower. The effect of the degree of facial deformity on PA therefore requires a large sample study to obtain further information on differences.

### 4 Three-dimensional accuracy of Middle face had the best performance for facial deformity

The difference color image (Fig 3) of 3D comparison between the two test scanners and the reference scanner showed a qualitative analysis result: the 3D error was distributed relatively evenly in middle face and there were more regions of closed zero error (green color) existing. In addition, the positive (red) and negative (dark blue) maximum errors were generally away from middle face and tended to appear in eyebrow and lip areas.

The quantitative analysis by calculating mean and SD of the 3D error in each facial partition (Table 1) gave good data support to the result. It is well-known that the accuracy of a scanner is composed of two parts: mean and standard deviation (SD) of 3D errors. The mean value reflects the system error of the scanner, which is always called “Correctness.” SD reflects the dispersion degree of a dataset, which is always understood as “Precision,” “Stability,” “Reliability,” and “Repeatability” [29]. The 3D accuracy of a scanner is presented by mean and SD comprehensively. In this study, the 3dMD scanner had the best performance both in terms of mean and SD as 0.48 ± 0.08 mm with middle face, and FaceScan scanner got the highest SD 0.09 mm with middle face. It embodied a good “Precision” in middle face, both for 3dMD and FaceScan, and explained the relatively equal distribution of errors showing of the difference color image (Fig 3). Although the mean value of the upper face was the highest for FaceScan, the SD is the worst of three parts. In contrast, the 0.52 ± 0.09 mm with middle face was the best comprehensive accuracy performance. Thus, the 3D accuracy of middle face had the best performance for both facial scanners in our study.

This result may have been caused by two factors: firstly, the 10 facial deformities in this study may have had a minimal impact on the middle face appearance. Secondly, it may have been due to the data post-processing algorithm. The principle of structured light or stereophotography scanning method has a common characteristic in that post-processing by software is needed to do registration for multi-angle 3D data acquired from scanning. The 3dMD scanner captures four partial face models at a time, while the FaceScan scanner collects three. The registration algorithm is usually based on the ICP algorithm to overlay the partial face models by their common areas according to the shape feature. Thus, the final accuracy of a complete face model is jointly influenced by the scanning accuracy and the registration accuracy together. Optical scanning is good at dealing with flat and convex surfaces, but poor with concave surfaces, which is why we mentioned the lower accuracy with the lower face above. However, the registration (ICP) algorithm requires sufficient shape feature (curvature changes significantly) to improve its accuracy. Hence, the flat area, just like the Upper face, may be more difficult to register, while the nasal and zygomatic regions in the middle face could improve the algorithm’s accuracy.

### 5 The PA is lower than NA of both optical facial scanners

The NA of an optical scanner is usually acquired by measuring standard geometric models provided by manufacturers [30]. In general, it reflects the best measurement accuracy of the scanner. However, in clinical application, many factors can affect the performance and reduce the final accuracy of a facial scanner. They include skin texture (pore, fine hair), color, contrast, roughness, micro-movements (caused by muscle contraction, breathing, and psychological state) and facial deformities. Thus, the PA of a facial scanner has more reference value for clinical use.

We previously reported that the result from a study of the facial scanner’s PA for cast head model was 0.2 to 0.3 mm [14], and for normal face was 0.3 to 0.4 mm [15]. This study further reported the PA of facial scanner for 10 facial deformities patients from oral and maxillofacial surgery clinic. The results (Table 1) showed that PA for global face was about 0.5 to 0.6 mm, and approximately 0.6 to 0.7 mm in the deformed area. The two optical facial scanners had no statistical difference between each other for facial deformity in this study. All satisfied the clinical requirement (usually less than 1–2 mm deviation needed for soft-tissue 3D measurement) in oral hospitals.

## Conclusion

The results of this study demonstrated the following: The practical accuracies for optical facial deformity were approximately 0.5–0.6 mm with global face and 0.6–0.7 mm with deformed area. The 3D accuracy of different facial partitions is inconsistent for facial deformity; the middle face had the best performance in this study. There was no statistically significant difference in accuracy between the scanners based on the principle of stereophotography and structured light; they all satisfied oral clinic requirements. However, further research is needed on the PA performance of facial scanners for different facial deformity degrees, especially serious deformities.
